# The League of Mercy

**Published:** 1911-10-28

**Authors:** 


### The League of Mercy.

The address printed above was delivered on 23rd inst. to members of the League of Mercy and others at a meeting at 29 Portman Square, London, W. The meeting was crowded, a fact which bore eloquent testimony to the ability and zeal of Mrs. G. Lawson Johnston, the hostess, who is Lady President of the East Marylebone division of the League. Sir Herbert Beerbohm Tree presided, as the President of the division, and Lady Tree gave a very interesting address upon the names and statistics in the reports of the work done by the League in 1910. She made it clear that the report of the League of Mercy is a book to be studied by all those who have not yet lent a hand by becoming a member or an officer of the League. For there they will find the record of a great volume of personal service for the sick, which has the twofold merit (1) of producing the means for their succour, and (2) of giving a keen apprehension, to those whose names are recorded in it, of a helpful standard of daily life based upon the principles which led to the foundation of the League by King Edward VII.

The members pay their meed of praise for the blessings of health by giving personal service, deliberately, though it entails the raising of £1 a year each in small sums, with possibly contingent disagreeables. The League of Mercy gives to women of some age and experience the opportunity to render similar service as Vice-Presidents by using that experience to attract and win twenty members who should become imbued with the principle of personal service, for so they may be greatly helped in their daily life. Mrs. G. Lawson Johnston would welcome the names of some thirty men and women who desire to live rather than to exist, and are prepared to test conscientiously what the habitual rendering of a little personal service each year can do for them.

